# Supplementary material for: Metabolic Profiling of Pitaya (Hylocereus polyrhizus) during Fruit Development and Maturation
Source: Molecules. 2019 Mar 20;24(6):1114. doi: 10.3390/molecules24061114 (PMC6470951; doi:10.3390/molecules24061114)
Supplement: Supplementary file 1 [file molecules-24-01114-s001.pdf]

Supplementary material:

Table S1. Metabolites derived from GC-MS and LC–MS/MS.

Nob	RT (min)	Metabolites	m/z[M + H]$^+$	Main Fragments	References
		Amino Acids and Amines			
1	4.22	Alanine [a]	--	147, 73, 116, 79	STD and NIST
2	6.43	Acetamide [a]	--	220, 73, 79, 58, 147	NIST
3	6.57	Valine [a]	--	144, 73, 147, 79	STD and NIST
4	1.86	Leucine [b]	132	86, 55	Ref 57
5	8.73	proline [a]	--	73, 142, 79, 158, 52	STD and NIST
6	9.05	Glycine [a]	--	248, 174, 73, 79, 147,	STD and NIST
7	10.89	Serine [a]	--	100, 147, 204, 218	STD and NIST
8	11.81	Threonine [a]	--	73, 79, 117, 147	STD and NIST
9	12.97	Lysine [a]	--	290, 73, 174, 147, 79	NIST
10	16.46	Aspartic Acid [a]	--	232, 73, 147, 100, 156	STD and NIST
11	16.55	GABA [a]	--	304, 174, 73, 147, 86	STD and NIST
12	9.98	Glutamic Acid [a]	--	246, 73, 147, 128, 192	STD and NIST
13	23.81	1,4 -Butanediamine [a]	--	174, 73, 59	NIST
14	25.32	Glutamine [a]	--	147, 84, 102, 130	STD and NIST
15	1.39	Isoleucine [b]	132	86, 55	Ref 57
16	1.11	Arginine [b]	175	116, 60	Ref 57
17	6.13	Tryptamine [b]	161	144, 117, 91, 65	Ref 57
18	1.35	Tyramine [b]	138	121, 103	Ref 57
19	1.82	Tyrosine [b]	182	91, 136, 119	Ref 57
20	1.87	Methionine [b]	150	61, 104, 75, 81, 54	Ref 567
21	2.96	Phenylalanine [b]	166	120, 103,93, 72	Ref 57
22	5.67	Tryptophan [b]	205	146, 118, 188	Ref 57
		Sugars and Sugar Alcohols			
23	29.28	Fructose [a]	--	217, 103, 147, 133	STD and NIST
24	30.13	Glucose [a]	--	217, 147, 191, 204	STD and NIST
25	52.89	Sucrose [a]	--	361, 73, 103, 147, 217	STD and NIST
26	30.72	Sorbose [a]	--	319, 73, 147, 205, 103,	STD and NIST
27	23.17	Ribitol [a]	--	217, 73, 103, 147	NIST
28	28.03	Sorbitol [a]	--	259, 73, 147, 190, 129,	NIST
29	29.90	Mannose [a]	--	319, 73, 147, 103, 160,	NIST
30	35.99	Glycoside [a]	--	319, 73, 204, 147, 103,	NIST
31	52.43	Fucose [a]	--	333, 73, 117, 147, 219	NIST
32	52.19	Mannobiose [a]	--	361, 73, 204, 147, 129,	NIST
33	33.70	Glucopyranose [a]	--	217, 73, 191, 147, 129	NIST
34	27.24	Fructopyranose [a]	--	217, 73, 204, 147	NIST
35	29.85	Talopyranose [a]	--	73, 204, 191, 147, 217	NIST
36	26.99	Tagatofuranose [a]	--	257, 73, 217, 147	NIST
37	21.86	Xylose [a]	--	147, 91, 204, 217	NIST
38	38.44	Myo-Inositol [a]	--	147, 217, 305, 308	NIST
		Organic Acids and Fatty Acids			
39	15.37	Malic Acid [a]	--	189, 113, 175, 219	STD and NIST
40	14.69	Citramalic Acid [a]	--	247, 73, 75, 115, 147	STD and NIST
41	9.17	Succinic Acid [a]	--	249, 147, 73, 75, 79, 58	STD and NIST
42	10.23	Fumaric Acid [a]	--	127, 71, 69, 99, 56	STD and NIST
43	27.20	Citrus Acid [a]	--	347, 73, 147, 273	STD and NIST
44	28.75	Quinic Acid [a]	--	345, 73, 147, 75, 55	STD and NIST
45	19.64	Tartaric Acid [a]	--	292, 73, 147, 117	STD and NIST
46	27.89	2-ketoglutaric Acid [a]	--	245, 73, 147, 173, 55	NIST
47	34.76	Hexadecanoic Acid [a]	--	313, 73, 117, 132, 55	NIST

48	31.87	Glucuronic Acid [a]	--	333, 73, 147, 160, 189	NIST
49	46.49	Octadecanoic Acid [a]	--	341, 73, 117, 129, 55	NIST
50	47.96	9,12,15-Octadecatrienoic Acid [a]	--	319, 73, 147, 93, 207	NIST
51	10.82	2-Piperidinecarboxylic Acid [a]	--	156, 73, 79, 147	NIST
52	4.49	Oxalic Acid [a]	--	147, 73,133	STD and NIST
53	18.23	Threonic Acid [a]	--	292, 73, 147, 220, 117	NIST
		Betalains and Betalain Precursors			
54	4.53	Betanin [b]	551	389	Ref. 1
55	5.94	Isobetanin [b]	551	188, 389	Ref. 1
56	6.52	Phyllocatin [b]	637	593, 551, 389	Ref. 58
57	13.06	Betalamic Acid [b]	212,	168, 194, 105	Ref. 1
58	8.89	Hylocerenin [b]	695	651, 551, 389	Ref. 58
59	8.97	Quercetin 3-O-rutinoside [b]	611	303, 465	Ref. 57
60	17.43	Cyclo-5-O-glucoside [b]	358	340, 106, 88, 70, 57	Ref. 1
		Other Metabolites			
61	7.91	H_3PO_4 [a]	--	186, 174, 73, 147, 100,	NIST
62	18.95	Butane [a]	--	306, 73, 117, 147, 205	NIST
63	10.06	Vebonol [b]	453	322, 209, 114	Ref. 59
64	18.14	Sespendole [b]	520	184	Ref. 59
65	18.04	Vapiprost [b]	478	337	Ref. 59

Note: Metabolites [(a)], Metabolites derived from GC-MS. Metabolites [(b)], Metabolites derived from LC-MS/MS. STD, standard compound. Ref, reference. --, Data unlisted.

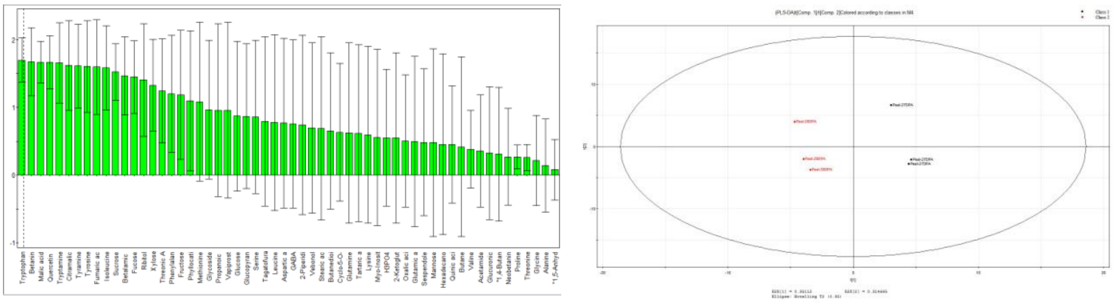

Figure S1. Peel-29DPA vs. Peel-27DPA.

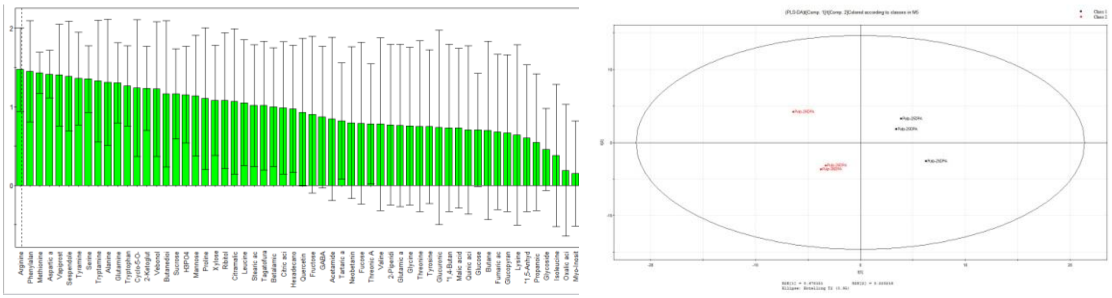

Figure S2. Pulp-26DPA vs. Pulp-25DPA.

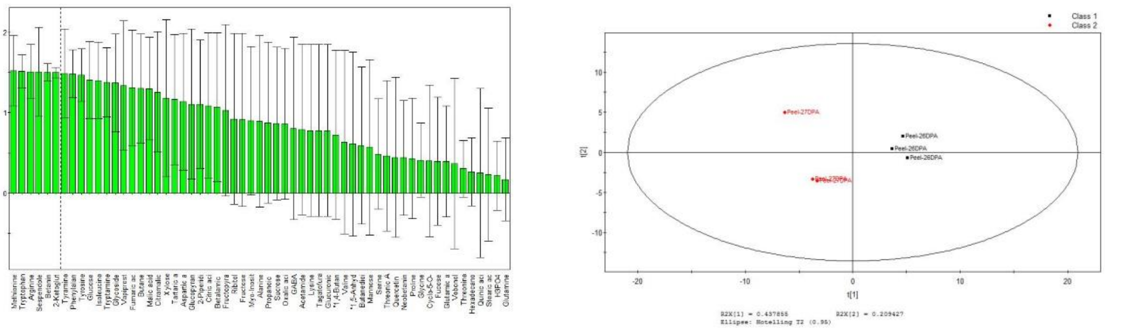

Figure S3. Pulp-26DPA vs. Pulp-27DPA.
